# Integrated modeling and analysis of intracellular and intercellular mechanisms in shaping the interferon response to viral infection

**DOI:** 10.1371/journal.pone.0186105

**Published:** 2017-10-11

**Authors:** Chunmei Cai, Jie Zhou, Xiaoqiang Sun, Tingzhe Sun, Weihong Xie, Jun Cui

**Affiliations:** 1 Key Laboratory of Gene Engineering of the Ministry of Education, State Key Laboratory of Biocontrol, School of Life Sciences, Sun Yat-sen University, Guangzhou, P. R. China; 2 Zhongshan School of Medicine, Sun Yat-sen University, Guangzhou, P. R. China; 3 Collaborative Innovation Center of Cancer Medicine, Sun Yat-sen University, Guangzhou, P. R. China; 4 AnQing Normal University, AnQing, PR China; University of Minnesota College of Veterinary Medicine, UNITED STATES

## Abstract

The interferons (IFNs) responses to viral infection are heterogeneous, while the underlying mechanisms for variability among cells are still not clear. In this study, we developed a hybrid model to systematically identify the sources of IFN induction heterogeneity. The experiment-integrated simulation demonstrated that the viral dose/type, the diversity in transcriptional factors activation and the intercellular paracrine signaling could strikingly shape the heterogeneity of IFN expression. We further determined that the IFNβ and IFNλ1 induced diverse dynamics of IFN-stimulated genes (ISGs) production. Collectively, our findings revealed the intracellular and intercellular mechanisms contributing to cell-to-cell variation in IFN induction, and further demonstrated the significant effects of IFN heterogeneity on antagonizing viruses.

## Introduction

The interferons (IFNs) are widely expressed by host cells to antagonize a variety of pathogens. There are three classes of IFN, specified type I–III, which are classified according to the similarity of their amino acid sequences and their receptor complexes [1, 2]. Type I IFN (IFNα, IFNβ, IFNω, IFNκ, and IFNε) and type III IFN (IFNλ1, IFNλ2, IFNλ3, and IFNλ4) play critical roles in antiviral responses [3, 4]. Pathogen recognition receptors, such as RIG-I and MDA5, recognize the invading viruses to initiate intracellular signaling cascades [5, 6], that leads to the activation of various transcriptional factors (TFs), including ATF2, c-Jun, NF-κB, IRF3 and IRF1 [7–10]. These TFs coordinately assemble an enhanceosome to trigger IFNs induction [11–13]. Secreted type I and III IFNs next bind to their own receptors to initiate JAK-STAT signaling, which eventually converges on ISGF3 to induce a large spectrum of IFN-stimulated genes (ISGs) production [14–16]. The establishment of antiviral state by multiple ISGs provides a powerful defense against viral replication and spread [17–20].

The activity of IFNs responses should be tightly controlled to antagonize viruses, since unwanted IFNs production is generally associated with immune dysfunction [21, 22]. Hence, an optimizing IFNs induction is required to orchestrate effective immune response. Previous studies showed that the variability in IFNs production deeply affects the subtle regulation of immune responses during viral infection [23–25]. In addition, those studies suggested the variability of IFNs temporal dynamics might result from several cell-intrinsic causes, including stochasticity in IFN gene expression [23, 25–28]. Regarding cell-extrinsic mechanisms, Chen *et a*l. and Zhao *et al*. identified that the variability of IFN induction was also due to viral property [26, 27]. Although it is conceivable that several factors might affect the dynamics of interferon, there is still lack of systematic analysis of underlying mechanisms in shaping variation of interferon response to viral infection. In addition, those studies mainly focused on heterogeneous production of type I IFN [23–29]. The heterocellular induction of type III IFN, which has been identified recently to play critical role in antiviral immunity [30, 31], has not been investigated yet. Moreover, pioneering researchers rarely focused on systematic investigation of the biological significance of variable IFNs induction [24].

Hence, in this study, we developed a hybrid stochastic–deterministic model to comprehensively investigate the dynamics of heterogeneous induction of IFNs triggered by RNA virus infection. By integrating the computational simulations and experiments, we aim to systematically investigate the intracellular and intercellular mechanisms for heterogeneity of IFNs induction. We further discussed the functional role of variation in IFN responses to viral infection by examining its effects on dynamics of ISGs to effectively antagonize virus.

## Material and methods

### Cell culture and reagents

HEK293T and A549 cells were cultured in DMEM medium (Hyclone) with 10% FBS (Gibco) incubated in a 5% CO_2_ chamber (Thermo Fisher Scientific). THP-1 cells were cultured in RPMI 1640 (Gibco) containing 10% FBS, which was also incubated in the 5% CO_2_ chamber. Cells were purchased from the Type Culture Collection of the Chinese Academy of Sciences (Shanghai, China). Before virus infection, HEK293T, THP-1 and A549 cells were planted in the density of 4 × 10^5^, 1 × 10^6^, 1.5 × 10^5^ cells/mL, respectively. Cells were then infected with VSV-eGFP or Sendai virus (SeV) in an indicated MOI (multiplicity of infection). A549 cells were treated with various concentrations of IFNβ (0.1 ng/ml, Peprotech) and IFNλ1 (20 ng/ml, Peprotech). The concentrations we adopted showed similar cytopathic effect reduction (CPER) of IFNβ and IFNλ1 as A. Meager *et a*/. [19] described.

### Immunoblot and antibodies

For Immunoblot, whole cell lysate was obtained with low-salt lysis buffer after virus infection for indicated time points. Protein samples were mixed with the 5X loading buffer (Cell Signaling Technology) and resolved by SDS-PAGE. After electrophoresis, protein was transferred to polyvinylidene fluoride membranes (Bio-Rad Laboratories) and then incubated with appropriate antibody. LumiGlo Chemiluminescent Substrate System (KPL) was used to detect specific band of certain protein. Antibodies used in immunoblot were listed as follows. Anti-IRF3 rabbit polyclonal antibody, goat anti–mouse IgG-HRP and goat anti–rabbit IgG-HRP antibodies were purchased from Santa Cruz Biotechnology Inc. Anti-phospho-IRF3 (Ser396) rabbit monoclonal antibody, anti-RIG-I rabbit monoclonal antibody, anti-p38 MAPK rabbit polyclonal antibody, anti-phospho-p38 MAPK (Thr180/Tyr182) rabbit polyclonal antibody, anti-SAPK/JNK rabbit polyclonal antibody, anti-phospho-SAPK/JNK (Thr183/Tyr185) rabbit polyclonal antibody, anti-TBK1/NAK (D1B4) rabbit monoclonal antibody, anti-phospho-TBK1/NAK (Ser172) rabbit monoclonal antibody, IκBα muse monoclonal antibody were purchased from Cell Signaling Technology. Anti-IRF1 mouse polyclonal antibody was bought from abcam^®^.

### RNA extraction and quantitative polymerase chain reaction(q-PCR)

Total RNA was extracted from cells with TRIzol reagent (Life Technologies), according to the manufacturer’s instructions. 1μg RNA was used to obtain cDNA through reverse transcription with HiScript® II Q RT SuperMix for qPCR (+gDNA wiper) kit (Vazyme). Quantitative real-time PCR was performed using Lightcycler 480 SYBR green I Master (Roche) with 2x superStar PCR Mix (GeneStar). The primers used in q-PCR were listed in Table 1.

**Table 1:** The primers list.

Genes	Sequences (5’-3’)
RPL13A	F: GCCATCGTGGCTAAACAGGTA R: GTTGGTGTTCATCCGCTTGC
IFNβ	F: CAGCAATTTTCAGTGTCAGAAGC R: TCATCCTGTCCTTGAGGCAGT
IFNλ1	F: CTTCCAAGCCCACCACAACT R: GGCCTCCAGGACCTTCAGC
ISG15	F:CGCAGATCACCCAGAAGATCG R:TTCGTCGCATTTGTCCACCA
ISG54	F:GGAGGGAGAAAACTCCTTGGA R:GGCCAGTAGGTTGCACATTGT
ISG56	F:TCAGGTCAAGGATAGTCTGGAG R:AGGTTGTGTATTCCCACACTGTA
Mx1	F:GTTTCCGAAGTGGACATCGCA R:CTGCACAGGTTGTTCTCAGC
Viperin	F:TGGGTGCTTACACCTGCTG R:GAAGTGATAGTTGACGCTGGTT
VSV	F:TGCAAGGAAAGCATTGAACAA R:GAGGAGTCACCTGGACAATCACT

### Generation of A549 knockout cell lines by CRISPR/Cas9

IRF1-, IRF3-, JNK1- as well as NF-κB p65-Cas9/small guide RNA (sgRNA) stably expressing A549 cells (bulk) were generated with the plenti-CRISPR V2, and the sequences of guide RNA (gRNA) were shown as follows:

IRF1 (5’-CACCGCTCGGATGCGCATGAGACCC-3’),
IRF3 (5’- CACCGGCACGCGCTTCCGCATCCCT-3’),
JNK1 (5’- CACCGTAGCTCTCTGTAGGCCCGCT-3’),
p65 (5’-CACCGGCGCTTCCGCTACAAGTGCG-3’).

### Computational modeling

We developed a hybrid mathematical model that couples deterministic ordinary differential equations (ODEs), describing the viral replication, signal transduction and ISGs production, and Gillespie algorithm [32] of stochastic simulation for IFNs’ gene transcription. For details in model description, please refer to S3 Appendix. The unknown parameters were estimated using nonlinear least square method using genetic algorithm. The table in S1 and S2 Appendices lists the detailed reactions and parameter values, respectively. The sensitivity coefficients of kinetic parameters were calculated to quantitatively evaluate critical parameters and components in the signaling pathways [33]. The simulation was performed in MATLAB R2012b (MathWork, Natwick, MA).

## Results

### Hybrid model could reproduce the dynamics of IFNs response to vesicular stomatitis virus infection

**Fig 1:**
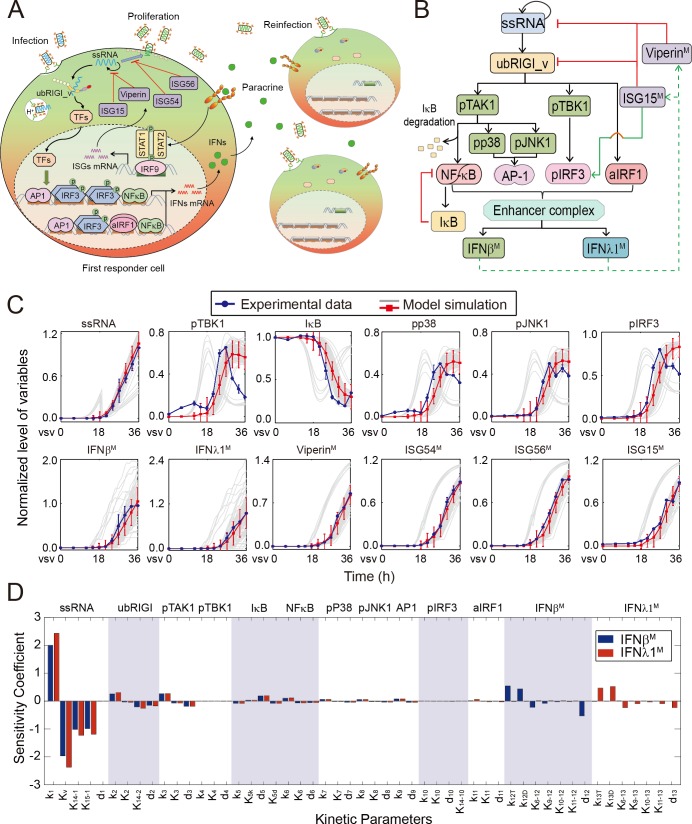
Mathematical model of IFN heterocellular induction by RNA viral infection. (A) Schematic representation of multi-cellular IFN response induced by RNA virus infection. (B) Detailed diagram of signaling pathways involved in IFNβ/λ1 response triggered by viral ssRNA. The variables with superscript M denoted its mRNA level. (C) Model simulations (red lines) fitted well with experimental data (blue dots) measured in A549 cells by VSV infection (MOI = 0.05). The gray lines denote 100 simulations randomly selected from 10,000 cells, and the red lines represent average level of 10,000 simulations. The data of both experiment and simulation are mean ± SD. The mean squared error (MSE) between the simulation and experimental data is 0.0112. (D) Local sensitivity analysis of *IFN*β and *IFN*λ*1* mRNA induction with respect to each kinetic parameter. The blue and red bars represent the sensitivity coefficients of IFNβ^M^ and IFNλ1^M^ respectively.

Upon vesicular stomatitis virus infection (VSV) treatment in A549 cells, we found that the response of IFNβ and IFNλ1 was the strongest among type I and III IFNs respectively, while there was almost no response of type II IFN (Figure A in S4 Appendix). Thus, we measured the induction of IFNβ and IFNλ1 to represent the antiviral response of type I and III IFN signaling pathway, respectively. According to previous studies and our experimental data, we concluded a schematic representation of multicellular IFNs response induced by RNA virus infection as shown in Fig 1A. A small number of host cells are infected by RNA viruses in early phase, and the invading viruses subsequently initiate self-replication [6]. Meanwhile, upon binding to viral ssRNA, RIG-I initiates multiple intracellular signaling cascades to evoke the activation of TFs, including NF-κB, AP1, IRF3 and IRF1, which assemble an enhanceosome complex to induce the induction of IFNβ and IFNλ1 [11–13]. IFNs are next secreted by first responder cells, triggering a wide set of ISGs expression to effectively antagonize viral infection [3, 23]. Besides, the newly assembled virus particles are released by budding to re-infect other host cells [6]. Fig 1B presents a schematic diagram of signaling pathways involved in IFNβ/λ1 response triggered by viral ssRNA.

To analyze mechanistically heterocellular dynamics of IFNs response to viral infection, we developed a hybrid model with deterministic and stochastic modules (S3 Appendix) (Fig 1A and 1B). To estimate the unknown parameters of model, we adopted nonlinear least square method to minimize the sum of squared differences between experimental and simulated data by employing genetic algorithm [34]. The dynamic patterns of simulations (Fig 1C) fitted well with experimental data (Figure A-D in S4 Appendix), and the mean squared error (MSE) was 0.0112. Therefore, our mathematical modeling could reproduce temporal patterns of variables involved in antiviral response. A local sensitivity analysis was performed to quantitatively calculate sensitivities of IFNβ^M^ (*IFN*β mRNA) or IFNλ1^M^ (*IFN*λ*1* mRNA) induction with respect to kinetic parameters. The higher absolute value of sensitivity coefficient indicates that the perturbed parameter is more critical to interferon responses. Our model simulations suggested that the expressions of IFNβ^M^ and IFNλ1^M^ were sensitive to parameters involved in virus replication and their induction (Fig 1D).

### Virus property significantly affects the variation of IFNs early expression

**Fig 2:**
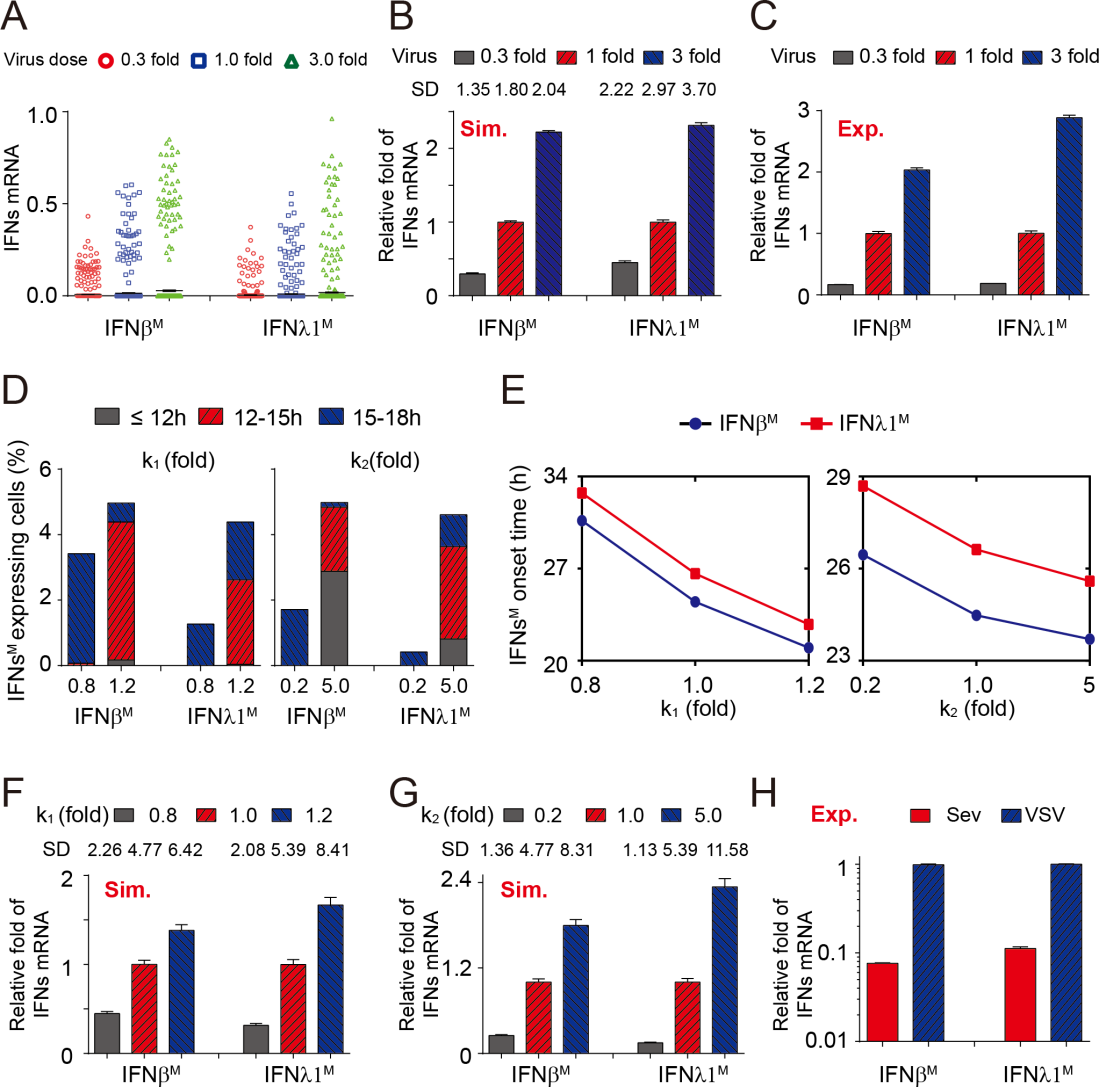
Viral properties affect the variation of IFNs early induction. (A-B) The early IFNβ/λ1^M^ induction with varying fold of viral dose treatment. (A) Each column includes 1,000 cells selected stochastically from 10,000 simulations (t = 18h). The red circle, blue square and green triangle denotes 0.3, 1.0 and 3.0 fold of initial viral dose respectively. (B) Each bar includes 10,000 simulations (t = 24h). The gray, red and blue bars denote 0.3, 1.0 and 3.0 fold of viral dose respectively. Results are mean ± SEM. The standard deviation (SD) indicates the variation of IFNβ^M^ or IFNλ1^M^ induction among multicellular population. “Sim.” represents simulations by model. (C) Experimentally measured IFNβ/λ1 responses in A549 cells with VSV at a MOI of 0.3 (gray), 1.0 (red) or 3.0 (blue) fold of 0.05 (t = 24h). “Exp.” represents experimental data. The data are mean ± SD, n = 3. (D-E) Temporal variation in cellular IFNβ/λ1^M^ induction. The viral replication (parameter k_1_, left panel in D and E) and its ability to initiate anti-viral signal (parameter k_2_, right panel in D and E) significantly shapes the IFNs^M^ onset. (D) Virus affects temporal variability of early IFNs induction. The gray, red and blue module indicates the time interval in which the cellular IFNβ^M^ or IFNλ1^M^ expression onset occurs. (E) Viral properties control onset times of IFNβ^M^ (blue) and IFNλ1^M^ (red). Data are mean ± SEM, n = 10,000. (F-G) Viral properties modulate the variation of IFNs early induction. The gray, red and blue bars indicate 0.8, 1.0 and 1.2 fold of k_1_ respectively (F) or 0.2, 1.0 and 5.0 fold of k_2_ respectively (G). The results are mean ± SEM, n = 10,000, t = 18h. (H) Various types of viruses induce distinct IFNβ/λ1^M^ expression measured by q-PCR assay. The red and blue bars represent SeV and VSV respectively. The data are mean ± SD, n = 3.

We next investigated the impacts of viral property on the heterogeneity of IFN expression, and found that increasing the dose of invading viruses resulted in more skewed distribution of IFNs expression (Fig 2A) and greater values of standard deviation (SD), which was used to measure the dispersion of data (Fig 2B) [35, 36]. Thus, increasing viral dose could amplify the cell-to-cell variation of IFNs induction. Importantly, the simulated tendency of IFNs response with increasing stimuli was consistent with experimental data (Fig 2B and 2C). Diverse viruses might evoke different dynamics of IFN expression. The k_1,_ related to viral replication, and k_2_, related to viral ability to initiate anti-viral signal are closely related to virus type [37–40]. Our analysis revealed that the increase in value of k_1_ or k_2_ resulted in earlier onset of the IFNβ^M^ and IFNλ1^M^ (Fig 2D and 2E) as well as greater values of SD (Fig 2F and 2G), indicating that diverse virus types significantly affected variability in IFN induction among multiple cells. Moreover, the experimental results (Fig 2H) confirmed our model prediction (Fig 2F and 2G) that various types of viruses might initiate distinct inductions of IFNβ^M^ and IFNλ1^M^. Collectively, these results demonstrated that the dose and type of viruses could dramatically modulate the cell-to-cell variability in IFNs induction among multicellular population.

### The diversity in TFs activation shapes the heterogeneity of IFNs early dynamics

**Fig 3:**
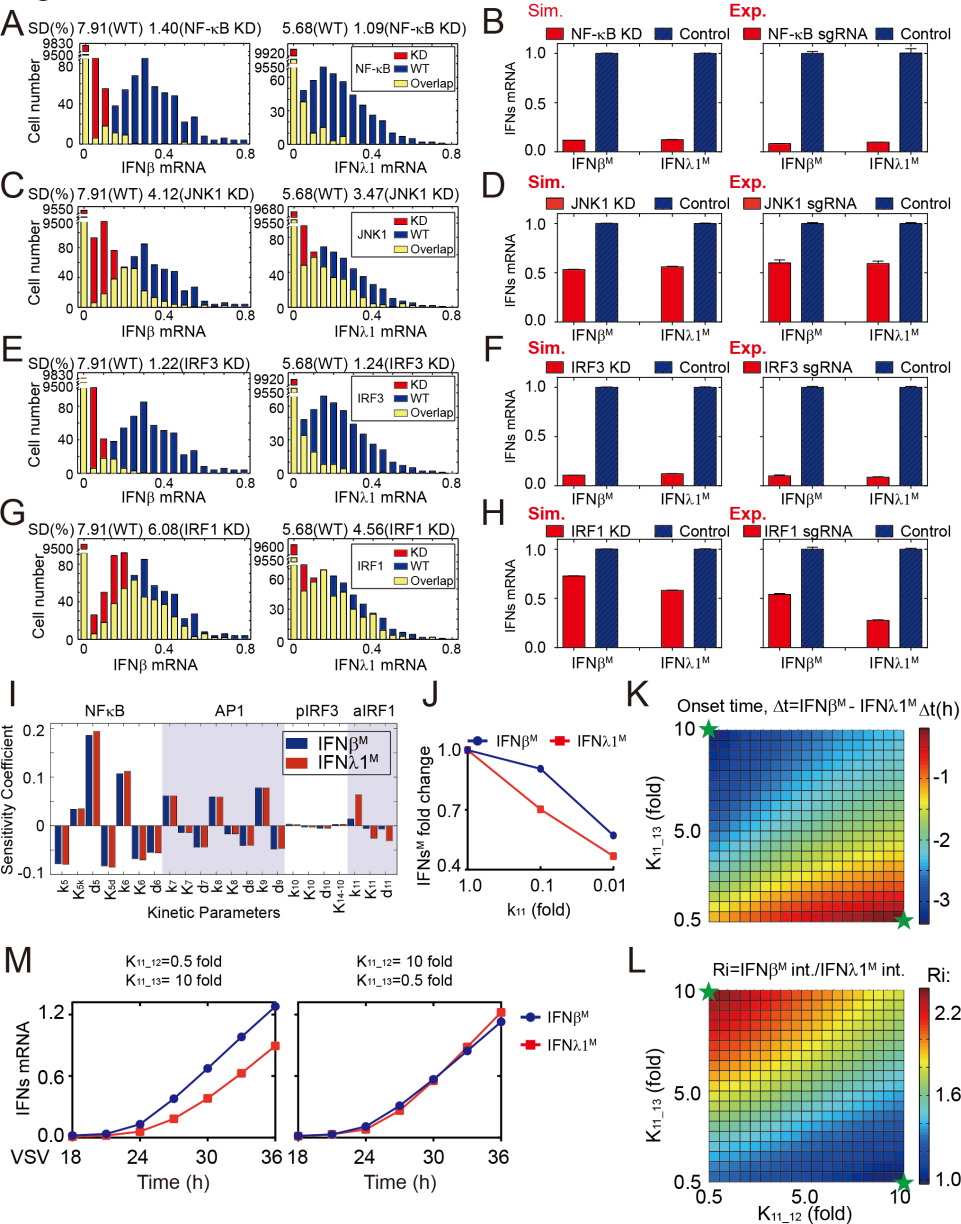
The variety among TFs activation significantly affects the variation and magnitude of IFNs response. (A, C, E and G) Distributions of IFNβ^M^ and IFNλ1^M^ levels. The knockdown (KD, red bars) of initial levels of NF-κB (A), JNK1 (C), IRF3 (E) and IRF1 (G) have significant effects on cell-to-cell variation of IFNs expression compared to wild type (WT, blue bars), where the yellow bars indicate the overlap between WT and KD. (B, D, F and H) The knockdown (KD, red bars) of initial levels of NF-κB (B), JNK1 (D), IRF3 (F) and IRF1 (H) reduce the expressions of IFNβ/λ1^M^ through simulations (left panel) and experiments (right panel), compared to WT (blue bars). (I) Local sensitivity analysis of integrated output of IFNβ^M^ and IFNλ1^M^ induction with respect to kinetic parameters involved in TFs activation. The blue and red bars denote IFNβ^M^ and IFNλ1^M^ respectively. (J) The change of IRF1 activation rate (k_11_) more greatly impacts IFNλ1^M^ than IFNβ^M^. The blue circle and red square indicate fold changes of IFNβ^M^ and IFNλ1^M^ respectively. (K-L) The change of K_11_12_ or K_11_13_ affects the difference between IFNβ^M^ and IFNλ1^M^ in (K) onset-time (Δt), and (L) integrated values (Ri). (M) The ratio between K_11_12_ and K_11_13_ affects the variety between temporal dynamics of IFNβ^M^ and IFNλ1^M^. The blue and red lines denote IFNβ^M^ and IFNλ1^M^ respectively. These two sets of K_11_12_ and K_11_13_ values were referred to green stars in (K) and (L) respectively. The data are mean ± SEM, n = 10,000.

The activation of TFs including NF-κB, AP1, IRF3 and IRF1, induced by viral infection, coordinately and cooperatively trigger IFNβ or IFNλ1 induction [11–13]. To investigate the function of multiple TFs activation on IFNs induction, we reduced the concentration of NF-κB, JNK1, IRF3 or IRF1 via reducing initial values of variables in our mathematical model and conducting the knockdown assay through CRISPR/Cas9 technology (Figure A in S5 Appendix). The simulated results showed that the down-regulation of each TF mentioned above resulted in reduction of both abundance (the distribution of IFNs move from high to low values) and cell-to-cell variation (the SD shifts from 7.91 to 1.40, 4.12, 1.22 or 6.08 percent for IFNβ^M^, and from 5.68 to 1.09, 3.47, 1.24 or 4.56 percent for IFNλ1^M^) of IFNs induction among 10,000 cells (Fig 3A, 3C, 3E and 3G and Figure B in S5 Appendix). Besides, both simulations and experiments suggested that the activation of NF-κB (Fig 3A and 3B) and IRF3 (Fig 3E and 3F) were crucial for strength and variability of IFNs induction. When reduced the initial amount of NF-κB, JNK1 or IRF3, the inhibitory effect on IFNβ^M^ expression was similar to IFNλ1^M^ (Fig 3B, 3D and 3F). However, reducing the level of IRF1 resulted in a stronger inhibitory effects on IFNλ1^M^ than IFNβ^M^ expression (Fig 3H). This was consistent with the local sensitivity analysis (Fig 1D), which indicated that IFNβ^M^ and IFNλ1^M^ expression have similar sensitivity to parameters perturbations involved in NF-κB, AP1 or IRF3 activation, while IFNλ1^M^ induction was much more sensitive to parameters related to IRF1 activation than IFNβ^M^ (Fig 3I). Moreover, a detailed model analysis also demonstrated that the change of IRF1 activation rate (k_11_) had differential impacts on IFNβ and IFNλ1 induction, while others did not (Fig 3J and Figure C in S5 Appendix). We then hypothesized that the difference between IFNβ and IFNλ1 promoter binding affinity with aIRF1 (K_11_12_ and K_11_13_ respectively) might be a potential cause of distinct effects of aIRF1 on IFNβ and IFNλ1 production. The *in silico* simulation showed that varying the value of K_11_12_ and/or K_11_13_ seriously affected the difference between IFNβ and IFNλ1 induction including onset time, integral value and dynamic patterns (Fig 3K–3M). In summary, these results demonstrated that the variety among intracellular TFs activation could affect the variability of IFNs early induction, and that the distinct influence of aIRF1 on IFNβ and IFNλ1 induction might arise from asymmetric binding affinities of IFNβ and IFNλ1 promoters to aIRF1.

### IFN paracrine by first responder cells shapes the heterogeneity of IFN responses among multicellular population

**Fig 4:**
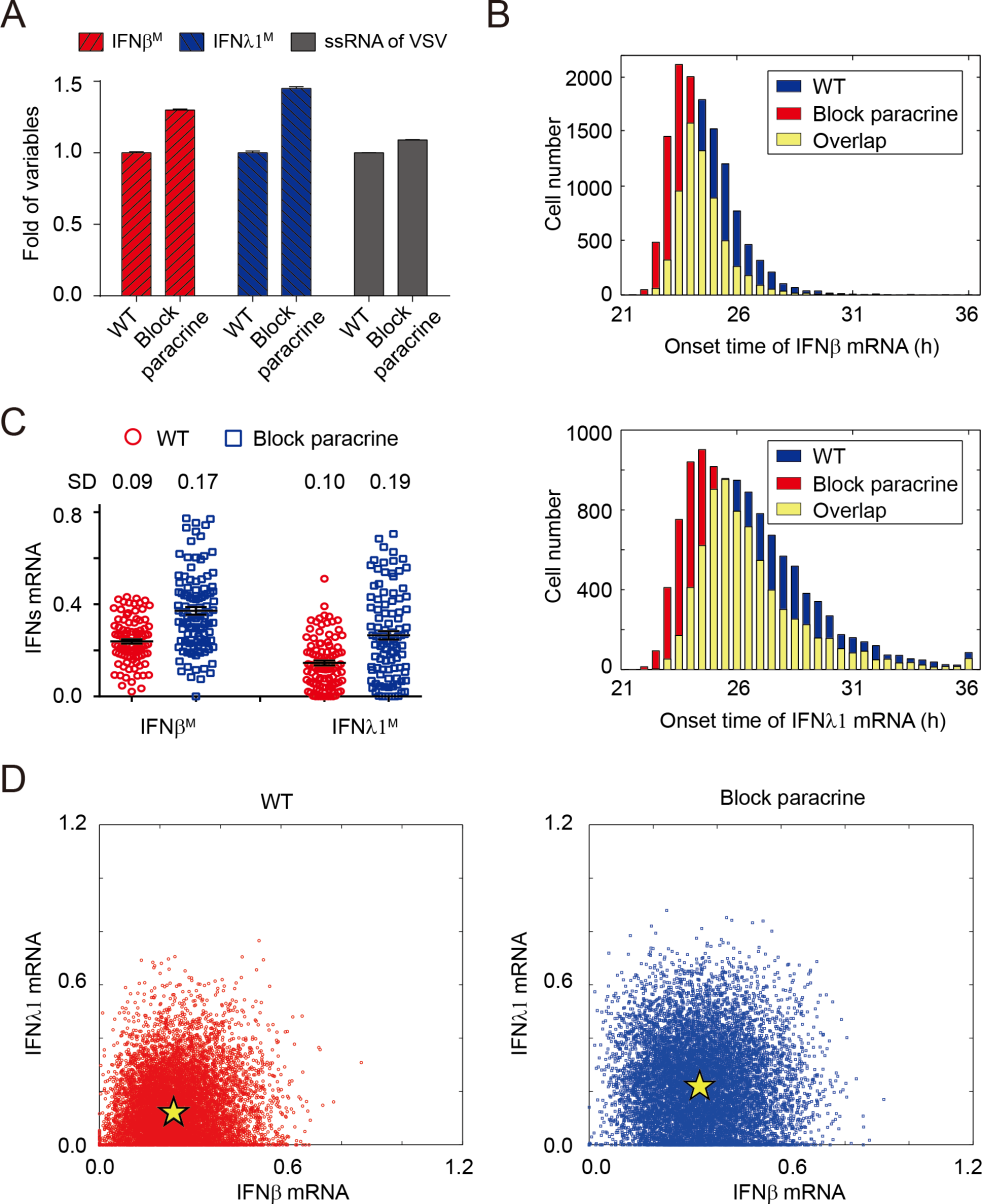
The paracrine of IFNs impacts the viral replication and cellular variation of IFN responses at late phase of viral infection. (A) The function of IFN paracrine signaling on IFNs response and viral replication (n = 10,000, t = 27h). The red, blue and gray bars denote simulated levels of IFNβ^M^, IFNλ1^M^ and ssRNA, respectively. The data are mean ± SEM. (B) The paracrine secretion of IFNs influences the temporal onset of IFNβ^M^ and IFNλ1^M^ response during late phase of viral infection (n = 9,500). The red, blue and yellow bars indicate the wild-type, paracrine blockage and overlap. (C) Paracrine blockage has significant effects on cell-to-cell variation of IFN expression. Each column includes 100 cells selected stochastically from 9,500 simulations infected at late phase. Data are shown as mean ± SEM, t = 27h. (D) The dispersion of IFNs might dramatically increase in paracrine blockage (right panel, blue squares) compared to that under wild-type condition (left panel, WT, red circles) (n = 9,500, t = 27h).

The paracrine of IFNs secreted by early infected cells may amplify the antiviral effects at the late stage of immune response via inducing the expression of a large spectrum of ISGs [3, 23]. Regarding to our hybrid model, several selected ISGs (e.g. Viperin) act to suppress the IFNs response by inhibiting the replication of ssRNA, which creates negative feedback loops in IFNs signaling. To check how intercellular paracrine modulates IFNs response to viral infection, we implemented the computational approach to block IFNs paracrine via abolishing the IFNs diffusion, which allow the cell to be only activated by the IFNs secreted by itself. Blocking the paracrine signaling might significantly increase the level of viral ssRNA, *IFN*β mRNA and *IFN*λ*1* mRNA (Fig 4A), indicating that the intercellular paracrine of IFNs plays a critical role in suppressing the viral replication to avoid excessive production of IFNs among multicellular population. The simulation analysis also demonstrated that blocking the paracrine resulted in earlier onset of IFNs (the distribution of IFNs onset time moved from high to low) (Fig 4B), which suggested that the paracrine of IFNs could inhibit the viral replication at the late stage of virus infection to weaken the IFN signaling through negative feedback loops. We further focused on the function of negative feedback loops generated by intercellular paracrine on cell-to-cell variability in IFNs expression. Our findings illustrated that blocking paracrine signaling increased the value of SD in both *IFN*β mRNA and *IFN*λ*1* mRNA (Fig 4C). In addition, the scatter diagram also showed that the distribution of IFNβ^M^ and IFNλ1^M^ was more dispersed in mutated cells (block paracrine) than wild-type (WT) cells (Fig 4D). Therefore, the negative feedback loops generated by intercellular paracrine could restrain the variability of IFN dynamic induction at the later stage of viral infection.

### IFNβ and IFNλ1 induce distinct dynamics of ISGs to antagonize viruses

**Fig 5:**
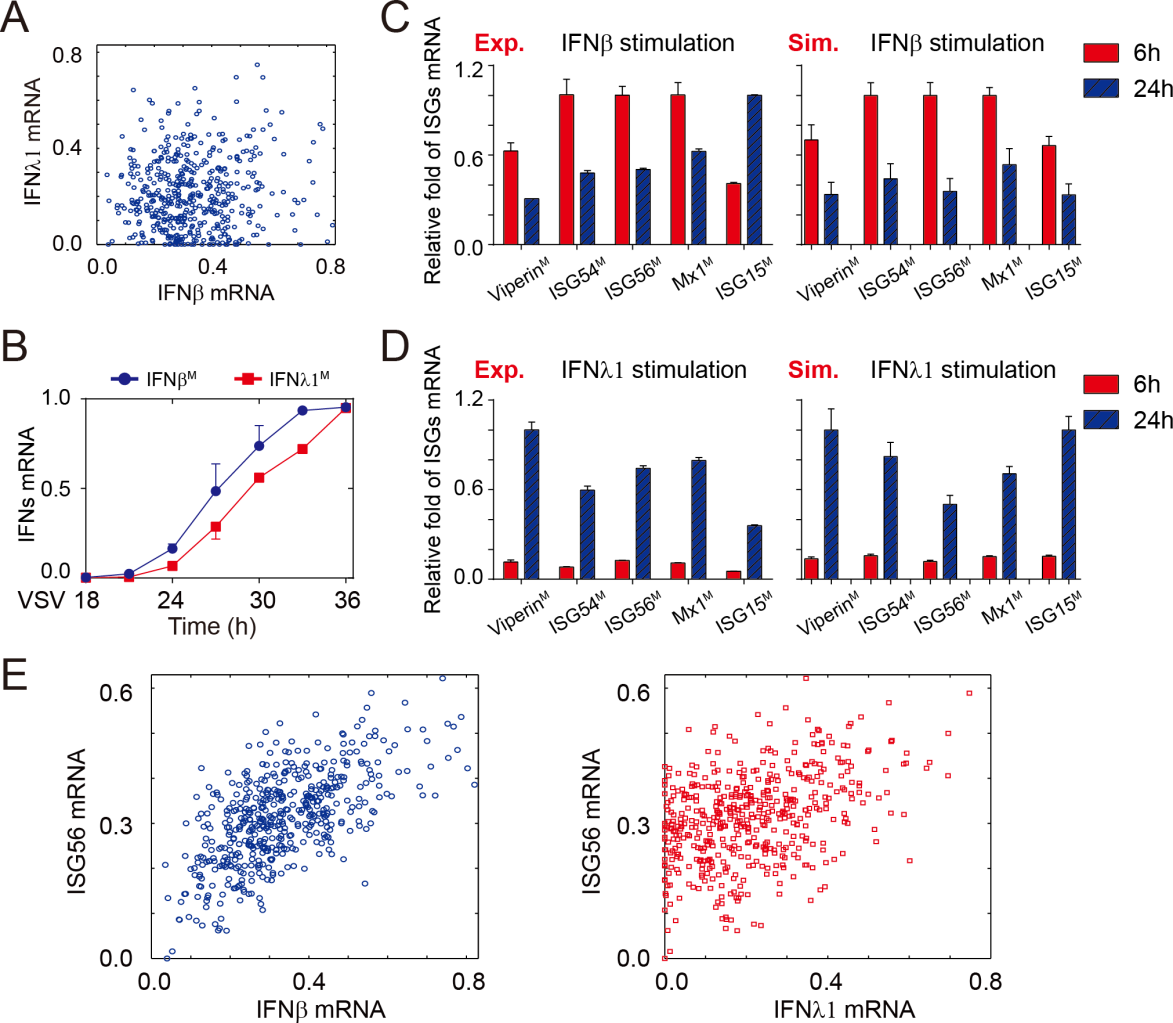
The IFNβ and IFNλ1 induce diverse temporal patterns of ISGs to antagonize virus. (A) Scatter plot of the distribution of *IFN*β and *IFN*λ*1* mRNA in early infected cells (n = 500, t = 18h). (B) Time courses of differential expressions of IFNβ^M^ and IFNλ1^M^. The blue and red lines denote IFNβ^M^ and IFNλ1^M^ respectively. The data are mean ± SEM, n = 3. (C-D) Induction of antiviral genes stimulated by IFNβ (C) and IFNλ1 (D). The red and blue bars represent gene expression after 6 and 24 hours of stimulation respectively. Data in these figures were presented as the mean ± SD of three independent experiments or 10,000 simulations. (E) The differential influences of IFNβ^M^ (left) and IFNλ1^M^ (right) on temporal pattern of ISG56^M^ by scatter analysis in early infected cells (n = 500, t = 18h). The squares of the Pearson correlation coefficients between ISG56^M^ and IFNβ/λ1^M^ (r^2^) are 0.40 and 0.16, respectively. The P values of two panels are less than 0.0001.

Our *in silico* analysis showed that the induction of *IFN*β and *IFN*λ*1* mRNA in each cell was heterogeneous among early infected cells (Fig 5A), and the experimental kinetics of *IFN*β and *IFN*λ*1* mRNA were also different (Fig 5B and Figure A in S6 Appendix). To mechanistically analyze the co-existence of type I and type III IFNs systems in antiviral response, we integrated simulations and experiments to determine the effects of IFNβ/λ1 on ISGs gene program. We found that the IFNλ1 induced a delayed but profound expression of most ISGs during 24 hours post-infection, whereas IFNβ-triggered ISGs almost peaked early and then declined at 24 hours (Fig 5C and 5D). The scatterplot suggested that the correlation between early induction of ISGs and IFNβ was much stronger than IFNλ1 via comparing the Pearson correlation coefficients (r^2^) (Fig 5E and Figure B-E in S6 Appendix, the r^2^ are 0.40, 0.31, 0.34, 0.28 and 0.33 for IFNβ^M^, and 0.16, 0.29, 0.22, 0.22 and 0.21 for IFNλ1^M^). Taken together, these results suggested that the heterogeneity of co-expressing *IFN*β and *IFN*λ*1 *evokes distinct dynamics of ISGs expression to provide complicated and redundant immune response to virus infection.

## Discussion

This study employed a systems biology approach to investigate the intra-/extra-cellular mechanisms of cell-to-cell variation in IFNs induction and its functional effects during antiviral immune response. To comprehensively and systematically investigate the heterogeneity of IFNs early induction, we developed a hybrid model coupling a deterministic module describing virus-induced signal transduction and ISGs production and a stochastic module describing viral infection and IFNβ/λ1 expression. Our model captured the key kinetics of the molecular and cellular signals in IFN induction.

Through mathematical modelling, we demonstrated that the temporal distributions of IFNs production were dispersive (SD = 7.91% or 5.68% for IFNβ^M^ (t = 18h) or IFNλ1^M^ (t = 18h), respectively) (Fig 3A), which suggested that the IFNs responses were heterogeneous, consistent with previous studies [23–25]. The causes of heterocellular IFNs induction might originate from two sources: (i) cell-extrinsic factors, such as viral property (e.g., intensity or type of virus), and (ii) cell-intrinsic noise, arising from stochastic signal transduction and gene expression [23, 26–28]. In this study, we calculated the SD of IFN induction among whole population to denote the variability in IFN response. Upon varying the viral dose or value of k_1/2_, related to type of virus, the SD of IFNs induction significantly changed. These results suggested that the viral property could seriously modulate the heterogeneity of IFNs expression. In addition, our work showed that the diversity in TFs activation and paracrine signaling could seriously shape the cell-to-cell variation of IFN induction. In brief, our work revealed how the intercellular and intracellular mechanisms cooperatively shape the dynamics and variability of IFN response to viral infection.

Until now, the previous studies about IFNs heterogeneity mainly focused on type I IFN-, but not type III IFN-response to viral infection [23, 25–29, 41]. In addition, several studies have identified the effects of IRF3 and NF-κB activation on heterogeneity of IFNs [23, 41], while the function of IRF1 and AP1 signaling pathways on temporal variability of IFNs is still not clear. Our experimental and simulation results revealed that the diversity in TFs activation dramatically affected the variation of IFNβ/λ1 induction, and that the IRF3 and NF-κB were essential for IFNβ/λ1 response. Interestingly, the activation of IRF1 had distinct effects on IFNβ/λ1 expression, which provided a potential intracellular modulation for host to selectively initiate diverse dynamics of IFNβ/λ1 response to various pathogen challenge. We further revealed that the differential binding affinities of IFNβ/λ1 promoter with IRF1 might be responsible for distinct impacts of aIRF1 on IFNβ/λ1 response. Furthermore, our findings determined that IFNβ triggered more rapid induction of ISGs compared with IFNλ1, which was consistent with previous studies [2, 20, 42].

Collectively, our study demonstrated that the viral property, diversity in TFs activation and the paracrine signal provided intracellular and intercellular mechanisms in shaping the heterogeneity of IFNs response to viral infection. In addition, our model analysis attributed the distinct impacts of IRF1 activation on IFNβ and IFNλ1 expression to differential binding affinities between promoters and IRF1. Moreover, our results revealed that the IFNβ/λ1 induced diverse kinetics of ISGs production in individual cells to robustly antagonize viral infection. Our study provides mechanistic and functional insights into variation in IFNs response to virus stimuli, and advances our understanding on IFNs mediated immune responses.

## Supporting information

S1 Appendix Reactions and rates of the kinetic model. (PDF)

S2 Appendix Values of variables and parameters involved in the mathematical model. (PDF)

S3 Appendix Supplementary model formulation. (PDF)

S4 Appendix Temporal IFNs response to viral infection under various conditions. (A) Time-course of type I, II and III IFNs expression in A549 cells infected with VSV at a MOI of 0.05. (B-C) Real-time PCR analysis of VSV ssRNA, Viperin, ISG54, ISG56 and ISG15 mRNA in A549 cells infected with VSV at a MOI of 0.05 at indicated time points. (D) Cell lysates of A549 cells were collected at indicated time after VSV (MOI = 0.05) treatment by immunoblotting with the indicated antibodies. Data in these figures were presented as the mean ± SD of three independent experiments.
(TIF)

S5 Appendix Manipulating the activation of different transcriptional factors may evoke diverse IFNs expression. (A) KO efficiency of p65^-/-^, JNK1^-/-^, IRF3^-/-^ and IRF1^-/-^ A549 cell lines by CRISPR/Cas9 technology. (B) The scatters analysis of IFNs^M^ (t = 18h) under specified conditions. Each panel includes 10,000 simulations. (C) The change of NF-κB (left), JNK1 (middle) or IRF3 (right) activation rate (d5, k8 or k10) had similar effect on IFNβ^M^ and IFNλ1^M^. The blue circle and red square indicates IFNβ^M^ and IFNλ1^M^ fold change respectively.
(TIF)

S6 Appendix The diverse effects of IFNβ^M^ and IFNλ1^M^ on ISGs^M^ dynamics. (A) Real-time PCR analysis of IFNβ and IFNλ1 mRNA (IFNβ^M^ and IFNλ1^M^, respectively) in 293T, THP-1 and A549 cells infected with VSV at a MOI of 0.05. (B-E) The correlation between ISGs^M^ (B for Viperin, C for ISG54, D for Mx1 and E for ISG15) and IFNβ^M^ (left) and IFNλ1^M^ (right) by scatter analysis in early infected cells (n = 500, t = 18h). The squares of the Pearson correlation coefficients (r^2^) between Viperin^M^, ISG54^M^, Mx1^M^, ISG15^M^ and IFNβ^M^ are 0.31, 0.34, 0.28 and 0.33, respectively. In addition, r^2^ between Viperin^M^, ISG54^M^, Mx1^M^, ISG15^M^ and IFNλ1^M^ are 0.29, 0.22, 0.22 and 0.21, respectively. The P values of all panels are less than 0.0001.
(TIF)

## Abbreviations

VSV: vesicular stomatitis virus
SeV: Sendai virus
PIV5: parainfluenza virus type 5
NDV: Newcastle Disease Virus
IFN: interferon
ISGs: IFN-stimulated genes
sgRNA: small guide RNA
WT: wild-type
CPER: cytopathic effect reduction
ODEs: ordinary differential equations
MSE: mean squared error
SD: standard deviation
MOI: multiplicity of infection
q-PCR: quantitative polymerase chain reaction
