# Supplementary material for: Integrated modeling and analysis of intracellular and intercellular mechanisms in shaping the interferon response to viral infection
Source: PLoS One. 2017 Oct 11;12(10):e0186105. doi: 10.1371/journal.pone.0186105 (PMC5636135; doi:10.1371/journal.pone.0186105)
Supplement: S1 Appendix — (PDF) [file pone.0186105.s001.pdf]

S1 Table: Reactions and rates of the kinetic model.

	Reactions	Reaction rates
**1, Deterministic module:**		
R1	ssRNA -> 2·ssRNA	$\frac{k_1[ssRNA]}{K_V+[ssRNA]}\bullet\left(\frac{1}{K_{14\_1}+[ISG15^M]}+\frac{1}{K_{15\_1}+[Viperin^M]}\right)$
R2	ssRNA -> null	$d_1[ssRNA]$
R3	RIG-I + ssRNA -> ubRIG-I + ssRNA	$\frac{k_2[ssRNA](1-[ubRIG-I])}{(K_2+(1-[ubRIG-I]))(K_{14\_2}+[ISG15^M])}$
R4	ubRIG-I -> RIG-I	$d_2[ubRIG-I]$
R5	TAK1 + ubRIG-I -> pTAK1 + ubRIG-I	$\frac{k_3[ubRIG-I](1-[pTAK1])}{K_3+(1-[pTAK1])}$
R6	pTAK1 -> TAK1	$d_3[pTAK1]$
R7	TBK1 + ubRIG-I -> pTBK1 + ubRIG-I	$\frac{k_4[ubRIG-I](1-[pTBK1])}{K_4+(1-[pTBK1])}$
R8	pTBK1 -> TBK1	$d_4[pTBK1]$
R9	-> IκB_t	$\frac{k_5[NF-\kappa B]}{K_{5k}+[NF-\kappa B]}$
R10	IκB_t -> null	$\frac{d_5[pTAK1][I\kappa B\_t]}{K_{5d}+[I\kappa B\_t]}$
R11	IκB-NF-κB -> NF-κB	$\frac{k_6(1-[I\kappa B\_t])(1-[NF-\kappa B])}{K_6+(1-[NF-\kappa B])}$
R12	NF-κB -> IκB-NF-κB	$d_6[I\kappa B\_t][NF-\kappa B]$
R13	P38 + pTAK1 -> pP38 + pTAK1	$\frac{k_7[pTAK1](1-[pp38])}{K_7+(1-[pp38])}$
R14	pP38-> P38	$d_7[pp38]$
R15	JNK1 + pTAK1-> pJNK1 + pTAK1	$\frac{k_8[pTAK1](1-[pJNK1])}{K_8+(1-[pJNK1])}$

R16	pJNK1-> JNK1	$d_8[pJNK1]$
R17	pP38 + pJNK1 -> AP1	$k_9[pp38][pJNK1]$
R18	AP1 -> null	$d_9[AP1]$
R19	IRF3 + pTBK1-> pIRF3 + pTBK1	$\frac{k_{10}[pTBK1](1-[pIRF3])}{K_{10}+(1-[pIRF3])}$
R20	pIRF3-> IRF3	$\frac{d_{10}[pIRF3]}{K_{14\_10}+[ISG15^M]}$
R21	IRF1 + RIGIlag -> aIRF1 + RIGIlag	$\frac{k_{11}[RIGIlag](1-[aIRF1])}{K_{11}+(1-[aIRF1])}$
R22	aIRF1-> IRF1	$d_{11}[aIRF1]$
R23	-> ISG15^M	$\frac{k_{14b}[IFN\beta]}{K_{14b}+[IFN\beta]}+\frac{k_{14l}[IFN\lambda 1]}{K_{14l}+[IFN\lambda 1]}$
R24	ISG15^M -> null	$d_{14}[ISG15^M]$
R25	-> Viperin^M	$\frac{k_{15b}[IFN\beta]}{K_{15b}+[IFN\beta]}+\frac{k_{15l}[IFN\lambda 1]}{K_{15l}+[IFN\lambda 1]}$
R26	Viperin^M -> null	$d_{15}[\mathrm{Viperin}^M]$
R27	-> ISG54^M	$\frac{k_{16b}[IFN\beta]}{K_{16b}+[IFN\beta]}+\frac{k_{16l}[IFN\lambda 1]}{K_{16l}+[IFN\lambda 1]}$
R28	ISG54^M -> null	$d_{16}[\mathrm{ISG54}^M]$
R29	-> ISG56^M	$\frac{k_{17b}[IFN\beta]}{K_{17b}+[IFN\beta]}+\frac{k_{17l}[IFN\lambda 1]}{K_{17l}+[IFN\lambda 1]}$
R30	ISG56^M -> null	$d_{17}[\mathrm{ISG56}^M]$
R31	-> Mx1^M	$\frac{k_{18b}[IFN\beta]}{K_{18b}+[IFN\beta]}+\frac{k_{18l}[IFN\lambda 1]}{K_{18l}+[IFN\lambda 1]}$
R32	Mx1^M -> null	$d_{18}[\mathrm{Mx1}^M]$

2, IFNs gene transcription:

2.1 Deterministic description:

D1 NF-κB + AP1 + pIRF3 -> $IFN\beta^M$

$$k_{12T}\frac{[NF-\kappa B]}{K_{6\_12}+[NF-\kappa B]}\bullet\frac{[AP1]}{K_{9\_12}+[AP1]}\bullet\frac{[pIRF3]^4}{K_{10\_12}^4+[pIRF3]^4}$$

D2 NF-κB + AP1 + pIRF3 +aIRF1 -> $IFN\beta^M$

$$k_{12D}\frac{[NF-\kappa B]}{K_{6\_12}+[NF-\kappa B]}\bullet\frac{[AP1]}{K_{9\_12}+[AP1]}$$
$$\bullet\frac{[pIRF3]^2}{K_{10\_12}^2+[pIRF3]^2}\bullet\frac{[aIRF1]^2}{K_{11\_12}^2+[aIRF1]^2}$$

D3 $IFN\beta^M$ -> null

$$d_{12}[\mathrm{IFN}\beta^M]$$

D4 NF-κB + AP1 + pIRF3 -> $IFN\lambda 1^M$

$$k_{13T}\frac{[NF-\kappa B]}{K_{6\_13}+[NF-\kappa B]}\bullet\frac{[AP1]}{K_{9\_13}+[AP1]}\bullet\frac{[pIRF3]^4}{K_{10\_13}^4+[pIRF3]^4}$$

D5 NF-κB + AP1 + pIRF3 +aIRF1 -> $IFN\lambda 1^M$

$$k_{13D}\frac{[NF-\kappa B]}{K_{6\_13}+[NF-\kappa B]}\bullet\frac{[AP1]}{K_{9\_13}+[AP1]}$$
$$\bullet\frac{[pIRF3]^2}{K_{10\_13}^2+[pIRF3]^2}\bullet\frac{[aIRF1]^2}{K_{11\_13}^2+[aIRF1]^2}$$

D6 $IFN\lambda 1^M$ ->null

$$d_{13}[\mathrm{IFN}\lambda 1^M]$$

2.2 Stochastic description:

S1 NF-κB + AP1 + pIRF3 -> $IFN\beta^O$

$$k_{12T}\frac{[NF-\kappa B]}{K_{6\_12}+[NF-\kappa B]}\bullet\frac{[AP1]}{K_{9\_12}+[AP1]}$$
$$\bullet\frac{[pIRF3]^4}{K_{10\_12}^4+[pIRF3]^4}\bullet(2-[\mathrm{IFN}\beta^O])$$

S2 NF-κB + AP1 + pIRF3 +aIRF1 -> $IFN\beta^O$

$$k_{12D}\frac{[NF-\kappa B]}{K_{6\_12}+[NF-\kappa B]}\bullet\frac{[pIRF3]^2}{K_{10\_12}^2+[pIRF3]^2}$$
$$\bullet\frac{[AP1]}{K_{9\_12}+[AP1]}\bullet\frac{[aIRF1]^2}{K_{11\_12}^2+[aIRF1]^2}\bullet(2-[\mathrm{IFN}\beta^O])$$

S3 $IFN\beta^O$ -> null

$$d_{12}[\mathrm{IFN}\beta^O]$$

S4 NF-κB + AP1 + pIRF3 -> $IFN\lambda 1^O$

$$k_{13T}\frac{[NF-\kappa B]}{K_{6\_13}+[NF-\kappa B]}\bullet\frac{[AP1]}{K_{9\_13}+[AP1]}$$
$$\bullet\frac{[pIRF3]^4}{K_{10\_13}^4+[pIRF3]^4}\bullet(2-[IFN\lambda 1^O])$$

S5	NF-κB + AP1 + pIRF3 +aIRF1 -> IFNλ1^O	$k_{13D}\frac{[NF-\kappa B]}{K_{6\_13}+[NF-\kappa B]}\bullet\frac{[pIRF3]^2}{K_{10\_13}^2+[pIRF3]^2}\bullet\frac{[AP1]}{K_{9\_13}+[AP1]}\bullet\frac{[aIRF1]^2}{K_{11\_13}^2+[aIRF1]^2}\bullet(2-[IFN\lambda 1^O])$
S6	IFNλ1^O -> null	$d_{13}[\mathrm{IFN}\lambda 1^O]$
S7	IFNβ^O -> IFNβ^M	$a_{12}[\mathrm{IFN}\beta^O]$
S8	IFNβ^M -> null	$b_{12}[\mathrm{IFN}\beta^M]$
S9	IFNλ1^O -> IFNλ1^M	$a_{13}[\mathrm{IFN}\lambda 1^O]$
S10	IFNλ1^M -> null	$b_{13}[\mathrm{IFN}\lambda 1^M]$
