# Supplementary material for: Integrated modeling and analysis of intracellular and intercellular mechanisms in shaping the interferon response to viral infection
Source: PLoS One. 2017 Oct 11;12(10):e0186105. doi: 10.1371/journal.pone.0186105 (PMC5636135; doi:10.1371/journal.pone.0186105)
Supplement: S2 Appendix — (PDF) [file pone.0186105.s002.pdf]

S2 Table: Values of variables and parameters involved in the mathematical model.

Symbols	Description	Values	References
**1, Deterministic module:**			
$X1_0$	The normalized initial value of ssRNA of VSV in each cell	5.0947E-06	Measured
$X5_0$	The normalized initial value of total IκB (IκB_t) comprised by free IκB and IκB-NF-κB complex	0.9814	Measured
k_1	Rate constant of virus replication	0.0651	Fitted
K_v	Half-saturation constant of virus replication	0.4398	Fitted
K_{14_1}	Half-saturation constant of inhibition on virus replication by ISG15	0.5682	Fitted
K_{15_1}	Half-saturation constant of inhibition on virus replication by Viperin	0.5874	Fitted
d_1	Rate constant of ssRNA degradation	0.0010	Fitted
k_2	Rate constant of RIG-I ubquitination induced by ssRNA	0.0911	Fitted
K_2	Half-saturation constant of RIG-I ubquitination induced by ssRNA	0.1086	Fitted
K_{14_2}	Half-saturation constant of inhibition on RIG-I activation by ISG15	0.0314	Fitted
d_2	Rate constant of ubRIG-I inactivation	0.3486	Fitted
k_3	Rate constant of TAK1 phosphorylation induced by ubRIG-I	0.8831	Fitted
K_3	Half-saturation constant of TAK1 phosphorylation induced by ubRIG-I	0.2386	Fitted
d_3	Rate constant of pTAK1 inactivation	0.5623	Fitted
k_4	Rate constant of TBK1 phosphorylation induced by ubRIG-I	0.9998	Fitted
K_4	Half-saturation constant of TBK1 phosphorylation induced by ubRIG-I	0.1241	Fitted
d_4	Rate constant of pTBK1 inactivation	0.4051	Fitted
k_5	Rate constant of IκB_t inducible synthesis triggered by NF-κB	0.1235	Fitted
K_{5k}	Half-saturation constant of IκB_t inducible synthesis triggered by NF-κB	0.1622	Fitted
d_5	Rate constant of IκB_t degradation induced by pTAK1	0.7507	Fitted
K_{5d}	Half-saturation constant of IκB_t degradation induced by pTAK1	0.4098	Fitted
k_6	Rate constant of NF-κB activation releasing from IκB_t for the latter degradation	0.7506	Fitted
K_6	Half-saturation constant of NF-κB activation releasing from IκB_t for the latter degradation	0.9761	Fitted
d_6	Rate constant of NF-κB inactivation	0.6961	Fitted

k_7	Rate constant of p38 phosphorylation induced by pTAK1	0.8780	Fitted
K_7	Half-saturation constant of p38 phosphorylation induced by pTAK1	0.1471	Fitted
d_7	Rate constant of pp38 inactivation	0.4514	Fitted
k_8	Rate constant of JNK1 phosphorylation induced by pTAK1	0.8785	Fitted
K_8	Half-saturation constant of JNK1 phosphorylation induced by pTAK1	0.2059	Fitted
d_8	Rate constant of pJNK1 inactivation	0.4031	Fitted
k_9	Rate constant of AP1 formation	0.8653	Fitted
d_9	Rate constant of AP1 dissociation	0.4119	Fitted
k_{10}	Rate constant of IRF3 phosphorylation induced by pTBK1	0.9021	Fitted
K_{10}	Half-saturation constant of IRF3 phosphorylation induced by pTBK1	0.0947	Fitted
d_{10}	Rate constant of pIRF3 inactivation	0.6514	Fitted
K_{14_10}	Half-saturation constant of inhibition on pIRF3 inactivation by ISG15	0.9859	Fitted
k_{11}	Rate constant of IRF1 activation induced by delayed signal (RIGIlag) from ubRIG-I	0.9431	Fitted
K_{11}	Half-saturation constant of IRF1 activation induced by RIGIlag	0.4431	Fitted
d_{11}	Rate constant of aIRF1 inactivation	0.2512	Fitted
k_{14b}	Rate constant of $ISG15^M$ transcription induced by IFNβ	0.1160	Fitted
K_{14b}	Half-saturation constant of $ISG15^M$ transcription induced by IFNβ	0.1243	Fitted
k_{14l}	Rate constant of $ISG15^M$ transcription induced by IFN$\lambda 1$	0.1151	Fitted
K_{14l}	Half-saturation constant of $ISG15^M$ transcription induced by IFN$\lambda 1$	0.0939	Fitted
d_{14}	Rate constant of $ISG15^M$ degradation	0.1771	Fitted
k_{15b}	Rate constant of $Viperin^M$ transcription induced by IFNβ	0.1345	Fitted
K_{15b}	Half-saturation constant of $Viperin^M$ transcription induced by IFNβ	0.3060	Fitted
k_{15l}	Rate constant of $Viperin^M$ transcription induced by IFN$\lambda 1$	0.1106	Fitted
K_{15l}	Half-saturation constant of $Viperin^M$ transcription induced by IFN$\lambda 1$	0.2360	Fitted
d_{15}	Rate constant of $Viperin^M$ degradation	0.1347	Fitted
k_{16b}	Rate constant of $ISG54^M$ transcription induced by IFNβ	0.1619	Fitted

K_{16b}	Half-saturation constant of $ISG54^M$ transcription induced by IFNβ	0.1050	Fitted
k_{16l}	Rate constant of $ISG54^M$ transcription induced by IFNλ1	0.1093	Fitted
K_{16l}	Half-saturation constant of $ISG54^M$ transcription induced by IFNλ1	0.1431	Fitted
d_{16}	Rate constant of $ISG54^M$ degradation	0.2174	Fitted
k_{17b}	Rate constant of $ISG56^M$ transcription induced by IFNβ	0.2396	Fitted
K_{17b}	Half-saturation constant of $ISG56^M$ transcription induced by IFNβ	0.1050	Fitted
k_{17l}	Rate constant of $ISG56^M$ transcription induced by IFNλ1	0.1093	Fitted
K_{17l}	Half-saturation constant of $ISG56^M$ transcription induced by IFNλ1	0.1431	Fitted
d_{17}	Rate constant of $ISG56^M$ degradation	0.2742	Fitted
k_{18b}	Rate constant of $Mx1^M$ transcription induced by IFNβ	0.2132	Fitted
K_{18b}	Half-saturation constant of $Mx1^M$ transcription induced by IFNβ	0.0468	Fitted
k_{18l}	Rate constant of $Mx1^M$ transcription induced by IFNλ1	0.1264	Fitted
K_{18l}	Half-saturation constant of $Mx1^M$ transcription induced by IFNλ1	0.0571	Fitted
d_{18}	Rate constant of $Mx1^M$ degradation	0.2718	Fitted
**2, IFNs gene expression:**			
**2.1 Deterministic motif:**			
k_{12T}	Rate constant of $IFN\beta^M$ transcription induced by NF-κB, AP1 and pIRF3	0.1563	Fitted
k_{12D}	Rate constant of m IFNβ transcription induced by NF-κB, AP1, pIRF3 and aIRF1	0.1342	Fitted
K_{6_12}	Half-saturation constant of $IFN\beta^M$ transcription induced by NF-κB	0.1538	Fitted
K_{9_12}	Half-saturation constant of $IFN\beta^M$ transcription induced by AP1	0.0470	Fitted
K_{10_12}	Half-saturation constant of $IFN\beta^M$ transcription induced by pIRF3	0.1413	Fitted
K_{11_12}	Half-saturation constant of $IFN\beta^M$ transcription induced by aIRF1	0.0706	Fitted
d_{12}	Rate constant of $IFN\beta^M$ degradation	0.1732	Fitted
k_{13T}	Rate constant of $IFN\lambda1^M$ transcription induced by NF-κB, AP1 and pIRF3	0.0675	Fitted
k_{13D}	Rate constant of m IFNλ1 transcription induced	0.0869	Fitted

	by NF-κB, AP1, pIRF3 and aIRF1		
K_{6_13}	Half-saturation constant of IFNλ1^M transcription induced by NF-κB	0.1542	Fitted
K_{9_13}	Half-saturation constant of IFNλ1^M transcription induced by AP1	0.0471	Fitted
K_{10_13}	Half-saturation constant of IFNλ1^M transcription induced by pIRF3	0.1421	Fitted
K_{11_13}	Half-saturation constant of IFNλ1^M transcription induced by aIRF1	0.1486	Fitted
d_{13}	Rate constant of IFNλ1^M degradation	0.0589	Fitted

2.2 Stochastic motif:

k_{12T}	Rate constant of IFNβ^O formation including NF-κB, AP1 and pIRF3	0.1563	Fitted
k_{12D}	Rate constant of IFNβ^O formation including NF-κB, AP1, pIRF3 and aIRF1	0.1342	Fitted
K_{6_12}	Half-saturation constant of IFNβ^O formation induced by NF-κB	0.1538	Fitted
K_{9_12}	Half-saturation constant of IFNβ^O formation induced by AP1	0.0470	Fitted
K_{10_12}	Half-saturation constant of IFNβ^O formation induced by pIRF3	0.1413	Fitted
K_{11_12}	Half-saturation constant of IFNβ^O formation induced by aIRF1	0.0706	Fitted
d_{12}	Rate constant of IFNβ^O dissociation	0.1732	Fitted
k_{13T}	Rate constant of IFNλ1^O formation including NF-κB, AP1 and pIRF3	0.0675	Fitted
k_{13D}	Rate constant of IFNλ1^O formation including NF-κB, AP1, pIRF3 and aIRF1	0.0869	Fitted
K_{6_13}	Half-saturation constant of IFNλ1^O formation induced by NF-κB	0.1542	Fitted
K_{9_13}	Half-saturation constant of IFNλ1^O formation induced by AP1	0.0471	Fitted
K_{10_13}	Half-saturation constant of IFNλ1^O formation induced by pIRF3	0.1421	Fitted
K_{11_13}	Half-saturation constant of IFNλ1^O formation induced by aIRF1	0.1486	Fitted
d_{13}	Rate constant of IFNλ1^O dissociation	0.0589	Fitted
a_{12}	Rate constant of IFNβ^M transcription	0.0918	[1]
b_{12}	Rate constant of IFNβ^M degradation	0.010	[1]
a_{13}	Rate constant of IFNλ1^M transcription	0.0890	[1]
b_{13}	Rate constant of IFNλ1^M degradation	0.010	[1]

1. Puszynski K, Gandolfi A, d'Onofrio A The Pharmacodynamics of the p53-Mdm2 Targeting Drug

Nutlin: The Role of Gene-Switching Noise Plos Computational Biology 2014; 10.
