# Supplementary material for: Integrated modeling and analysis of intracellular and intercellular mechanisms in shaping the interferon response to viral infection
Source: PLoS One. 2017 Oct 11;12(10):e0186105. doi: 10.1371/journal.pone.0186105 (PMC5636135; doi:10.1371/journal.pone.0186105)
Supplement: S3 Appendix — (PDF) [file pone.0186105.s003.pdf]

Supplementary Model Formulation

Model Construction

Signaling transduction is often a fast biological process (in typical time units of seconds/minutes), whereas the gene expression is a relative slow process (in typical time units of hours) [1, 2]. To describe the multi-scale regulations from signaling pathway to gene expression involved in the early variation of IFNs, we developed a hybrid model coupling a deterministic ODE module and a stochastic Gillespie module. Since the molecule numbers involved in viral replication, signal transduction and ISGs production are rather large, therefore we used deterministic ODEs to describe these processes [3]. It has also been reported that the process of IFNs gene expression is stochastic, so we adopted the Gillespie algorithm to elaborately simulate this process [4, 5]. Besides, it's well known that only a small number of host cells are infected in early phase, and some of these cells initiate the detectable antiviral responses [4, 6, 7]. To reduce complicity of model, we simply considered random infections of viruses and assumed normally distribution of infected viruses.

S1. Deterministic module

Vesicular stomatitis virus (VSV) invades the host cells, and initiates self-replication (reactions 1-2 in S1 Appendix) [8]. Meanwhile, upon binding to ssRNA of VSV, RIG-I activates mitochondrial antiviral signaling protein (MAVS, also known as IPS-1, VISA, and CARDIF) via CARD-CARD interaction to recruit downstream signaling-complexes, such as TAK1 and TBK1 [9-11]. To model these processes as simple as possible, we only consider the transduction from the activated RIG-I to the phosphorylation of TAK1 and TBK1 (reactions 3-8 in S1 Appendix). Subsequently, pTAK1 activates IKK complex to phosphorylate IκB, leading to ubiquitin (Ub)-mediated degradation of IκB and NF-κB activation; At the same time, pTAK1 induces phosphorylation of p38 and JNK1 which then phosphorylate ATF and ATF/c-Jun respectively to comprise AP1 [12-14]. In our model we assumed that pTAK1 phosphorylates IκB, p38 and JNK1 to activate transcriptional factors (TFs), including NF-κB and AP1 (reactions 9-18 in S1 Appendix). Besides, phosphorylated IRF3 is induced by pTBK1 to assemble its dimer form (reactions 19-20 in S1 Appendix) [15, 16]. When the antiviral response is initiated, the upstream signals primarily trigger nuclear IRF1 translocation leading to a large number of IRF1 activation and dimerization in cytoplasm [17]. A Three-hour delay from activated RIG-I to cytoplasmic IRF1 for latter translocation was assumed, specified as 'RIGIlag' (reactions 21-22 in S1 Appendix).

Upon VSV treatment in A549 cells, we found that some ISGs (data not shown) were induced by IFNs secreted by responsive cells via JAK-STAT pathway to efficiently antagonize virus [18]. We selected several representative ISGs, ISG15, Viperin, ISG54, ISG56 and Mx1, to further investigate their antiviral function. In addition, we assumed that the dynamic pattern of IFNβ and IFNλ1 proteins lagged one hour of '$IFN\beta^M$' and '$IFN\lambda 1^M$' (*IFNβ/λ1* mRNA) respectively (reactions 23-32 in S1 Appendix). It has been reported that ISG15 inhibits virus replication and promotes RIG-I degradation via ISGylation to negatively shape antiviral response, while ISG15 also ISGylates IRF3 to stabilize

activated IRF3 (reactions 1, 3, 20 in S1 Appendix) [19, 20]. Viperin has recently been identified to play a critical role in inhibiting viral replication (reactions 1 in S1 Appendix) [21]. Besides, Mx1, one of the first described inhibitors of virus entry, can inhibit re-infection [22]. As well, ISG54/56 have been indicated as regulators to suppress the initiation of viral translation [23]. Therefore, we assumed that Mx1 decreases initial quantity of virus during viral re-infection phase, while ISG54 and ISG56 reduce the total amount of viruses released from first responder cells.

The ordinary differential equations (ODEs) describing the dynamics of virus replication, signal transduction and ISGs production were given as follows:

$$\frac{\mathrm{d}[ssRNA]}{dt} = \frac{k_1[ssRNA]}{K_V + [ssRNA]} \bullet \left(\frac{1}{K_{14\_1} + [ISG15^M]} + \frac{1}{K_{15\_1} + [Viperin^M]} \right) - d_1[ssRNA] \tag{1}$$

$$\frac{\mathrm{d}[ubRIG-I]}{dt} = \frac{k_2[\mathrm{ss}RNA](1-[ubRIG-I])}{(K_2 + (1-[ubRIG-I]))(K_{14\_2} + [ISG15^M])} - d_2[ubRIG-I] \tag{2}$$

$$\frac{\mathrm{d}[pTAK1]}{dt} = \frac{k_3[ubRIG-I](1-[pTAK1])}{K_3 + (1-[pTAK1])} - d_3[pTAK1] \tag{3}$$

$$\frac{\mathrm{d}[pTBK1]}{dt} = \frac{k_4[ubRIG-I](1-[pTBK1])}{K_4 + (1-[pTBK1])} - d_4[pTBK1] \tag{4}$$

$$\frac{\mathrm{d}[I\kappa B\_t]}{dt} = \frac{k_5[NF-\kappa B]}{K_{5k} + [NF-\kappa B]} - \frac{d_5[\mathrm{p}TAK1][I\kappa B\_t]}{K_{5d} + [I\kappa B\_t]} \tag{5}$$

$$\frac{\mathrm{d}[NF-\kappa B]}{dt} = \frac{k_6(1-[I\kappa B\_t])(1-[NF-\kappa B])}{K_6 + (1-[NF-\kappa B])} - d_6[I\kappa B\_t][NF-\kappa B] \tag{6}$$

$$\frac{\mathrm{d}[pp38]}{dt} = \frac{k_7[pTAK1](1-[\mathrm{p}38])}{K_7 + (1-[p38])} - d_7[pp38] \tag{7}$$

$$\frac{\mathrm{d}[pJNK1]}{dt} = \frac{k_8[pTAK1](1-[pJNK1])}{K_8 + (1-[pJNK1])} - d_8[pJNK1] \tag{8}$$

$$\frac{\mathrm{d}[AP1]}{dt} = k_9[pp38][pJNK1] - d_9[AP1] \tag{9}$$

$$\frac{\mathrm{d}[pIRF3]}{dt} = \frac{k_{10}[pTBK1](1-[pIRF3])}{K_{10} + (1-[pIRF3])} - \frac{d_{10}[pIRF3]}{K_{14\_10} + [ISG15^M]} \tag{10}$$

$$\frac{\mathrm{d}[aIRF1]}{dt} = \frac{k_{11}[RIGIlag](1-[aIRF1])}{K_{11} + (1-[aIRF1])} - d_{11}[aIRF1] \tag{11}$$

$$\frac{\mathrm{d}[ISG15^M]}{dt} = \frac{k_{14b}[IFN\beta]}{K_{14b} + [IFN\beta]} + \frac{k_{14l}[IFN\lambda 1]}{K_{14l} + [IFN\lambda 1]} - d_{14}[ISG15^M] \tag{12}$$

$$\frac{d[\mathrm{Viperin}^M]}{dt} = \frac{k_{15b}[IFN\beta]}{K_{15b}+[IFN\beta]} + \frac{k_{15l}[IFN\lambda 1]}{K_{15l}+[IFN\lambda 1]} - d_{15}[\mathrm{Viperin}^M] \tag{13}$$

$$\frac{d[ISG54^M]}{dt} = \frac{k_{16b}[IFN\beta]}{K_{16b}+[IFN\beta]} + \frac{k_{16l}[IFN\lambda 1]}{K_{16l}+[IFN\lambda 1]} - d_{16}[ISG54^M] \tag{14}$$

$$\frac{d[ISG56^M]}{dt} = \frac{k_{17b}[IFN\beta]}{K_{17b}+[IFN\beta]} + \frac{k_{17l}[IFN\lambda 1]}{K_{17l}+[IFN\lambda 1]} - d_{17}[ISG56^M] \tag{15}$$

$$\frac{d[Mx1^M]}{dt} = \frac{k_{18b}[IFN\beta]}{K_{18b}+[IFN\beta]} + \frac{k_{18l}[IFN\lambda 1]}{K_{18l}+[IFN\lambda 1]} - d_{18}[Mx1^M] \tag{16}$$

S2. IFNs gene expression

Above TFs (NF-κB, AP1, IRF3 and IRF1) coordinately assemble an enhanceosome that binds to IFNs promoters to trigger their inductions, which is a hallmark of early antiviral responses against infection [24]. We first constructed a deterministic module for IFNs gene transcription to estimate kinetic parameters of integrated deterministic model using Genetic algorithm. Since we have mentioned above that the IFNs gene transcription is stochastic, we subsequently adopted a stochastic module via using Gillespie algorithm to accurately simulate the stochastic IFNs transcription.

S2.1. Deterministic description

The ODEs describing the dynamics of IFNs gene transcription are given as follows:

$$\begin{aligned}\frac{d[\mathrm{IFN}\beta^M]}{dt} &= k_{12T}\frac{[NF-\kappa B]}{K_{6\_12}+[NF-\kappa B]}\bullet\frac{[AP1]}{K_{9\_12}+[AP1]}\bullet\frac{[pIRF3]^4}{K_{10\_12}^4+[pIRF3]^4}\\ &+k_{12D}\frac{[NF-\kappa B]}{K_{6\_12}+[NF-\kappa B]}\bullet\frac{[AP1]}{K_{9\_12}+[AP1]}\bullet\frac{[pIRF3]^2}{K_{10\_12}^2+[pIRF3]^2}\bullet\frac{[aIRF1]^2}{K_{11\_12}^2+[aIRF1]^2}\\ &-d_{12}[\mathrm{IFN}\beta^M]\end{aligned} \tag{17}$$

$$\begin{aligned}\frac{d[\mathrm{IFN}\lambda 1^M]}{dt} &= k_{13T}\frac{[NF-\kappa B]}{K_{6\_13}+[NF-\kappa B]}\bullet\frac{[AP1]}{K_{9\_13}+[AP1]}\bullet\frac{[pIRF3]^4}{K_{10\_13}^4+[pIRF3]^4}\\ &+k_{13D}\frac{[NF-\kappa B]}{K_{6\_13}+[NF-\kappa B]}\bullet\frac{[AP1]}{K_{9\_13}+[AP1]}\bullet\frac{[pIRF3]^2}{K_{10\_13}^2+[pIRF3]^2}\bullet\frac{[aIRF1]^2}{K_{11\_13}^2+[aIRF1]^2}\\ &-d_{13}[\mathrm{IFN}\lambda 1^M]\end{aligned} \tag{18}$$

S2.2. Stochastic description

In our model there are 6 stochastic reactions, and the related propensities corresponding to the above ODEs model are listed below:

$$P1 = k_{12T}\frac{[NF-\kappa B]}{K_{6\_12}+[NF-\kappa B]}\bullet\frac{[AP1]}{K_{9\_12}+[AP1]}\bullet\frac{[pIRF3]^4}{K_{10\_12}^4+[pIRF3]^4}\bullet(2-[IFN\beta^O]) \tag{19}$$

$$P2 = k_{12D}\frac{[NF-\kappa B]}{K_{6\_12}+[NF-\kappa B]}\bullet\frac{[pIRF3]^2}{K_{10\_12}^2+[pIRF3]^2}\bullet\frac{[AP1]}{K_{9\_12}+[AP1]}\bullet\frac{[aIRF1]^2}{K_{11\_12}^2+[aIRF1]^2} \tag{20}$$

$$\bullet(2-[\mathrm{IFN}\beta^O])$$

$$P3 = d_{12}[\mathrm{IFN}\beta^O] \tag{21}$$

$$P4 = k_{13T}\frac{[NF-\kappa B]}{K_{6\_13}+[NF-\kappa B]}\bullet\frac{[AP1]}{K_{9\_13}+[AP1]}\bullet\frac{[pIRF3]^4}{K_{10\_13}^4+[pIRF3]^4}\bullet(2-[IFN\lambda1^O]) \tag{22}$$

$$P5 = k_{13D}\frac{[NF-\kappa B]}{K_{6\_13}+[NF-\kappa B]}\bullet\frac{[pIRF3]^2}{K_{10\_13}^2+[pIRF3]^2}\bullet\frac{[AP1]}{K_{9\_13}+[AP1]}\bullet\frac{[aIRF1]^2}{K_{11\_13}^2+[aIRF1]^2} \tag{23}$$

$$\bullet(2-[IFN\lambda1^O])$$

$$P6 = d_{13}[\mathrm{IFN}\lambda1^O] \tag{24}$$

In the above model, these 6 stochastic reactions describe the binding of NF-κB-AP1-IRF3 enhanceosome with promoters of IFNβ (P1) and IFNλ1 (P4), the binding of NF-κB-AP1-IRF3-IRF1 enhanceosome with promoters of IFNβ (P2) and IFNλ1 (P5), as well as the dissociation of enhanceosome from promoters of IFNβ (P3) and IFNλ1 (P6). We assumed that there are two gene copies of IFNβ and IFNλ1 respectively. Therefore, the number of $\mathrm{IFN}\beta^O$ or $\mathrm{IFN}\lambda1^O$, which denotes that the promoter of IFNβ or IFNλ1 was occupied by TFs enhanceosome, takes value randomly of 0, 1 or 2. The IFNs gene transcription is initiated after the TFs enhanceosome binds to promoters of IFNs to form IFNs^O, and the corresponding deterministic processes are listed below:

$$\frac{d[\mathrm{IFN}\beta^M]}{dt} = a_{12}[\mathrm{IFN}\beta^O]-b_{12}[\mathrm{IFN}\beta^M] \tag{25}$$

$$\frac{d[\mathrm{IFN}\lambda1^M]}{dt} = a_{13}[\mathrm{IFN}\lambda1^O]-b_{13}[\mathrm{IFN}\lambda1^M] \tag{26}$$

The hybrid simulation algorithm that couples the short-time scale reactions (ODE model) and long-time scale reactions (Gillespie algorithm) is described below:

(1). We calculate the length of time interval (τ) of reaction at long-time scale following a random exponential distribution with parameter λ, i.e.

$$\tau \sim Exp(\lambda),$$

where λ is the sum of above 6 propensities, $\lambda = \sum_{i=1}^{6} P_i$.

(2). During time interval [t, t+τ], we solve ODEs model (Equations (1-16 and 25-26)) at short-time scale.

(3). At time point $t+\tau$, we simulate the activation or deactivation of gene IFNβ and IFNλ using Random-Walk algorithm. More precisely, we generate a random number r from

the uniform distribution on [0, 1], and calculate intervals $I_1 = [0, P_1]$ and $I_i = \left(\sum_{j=1}^{i-1} P_j, \sum_{j=1}^{i} P_j \right]$ (*i*=2,..,6). Then by checking in which interval *r* is located, we determine the corresponding activation or deactivation of each gene.

(4). Finally, time $t+\tau$ replaces t. Go back to step (1) for next iteration until *t* is larger than T (36 hours in this work).

It has been reported that the extracellular diffusion of IFNs is really fast when compared to the timescale of IFNs response and expression [25]. Hence, we assumed the normal distribution of secreted IFNs. Following paracrine of IFNs, the binding of IFNs to their specific receptors amplifies the antiviral effects in late phase of immune response. In Fig 1C, we stimulated the A549 cells with VSV (MOI=0.05), and the experimental results demonstrated that most variables showed obvious changes only after 18 hours. Therefore, we assumed that the re-infection occurred at 18 hours following the VSV treatment in A549 cells. The mean of ssRNA at 18 hours under above conditions in early infected cells was approximately equal to 0.35. Therefore, we assumed that when the mean of ssRNA among early infected cells is above 0.35, the VSV may be released to invade uninfected cells.

Supplementary References

1. Puszynski K, Gandolfi A, d'Onofrio A The Pharmacodynamics of the p53-Mdm2 Targeting Drug Nutlin: The Role of Gene-Switching Noise Plos Computational Biology 2014; 10.
2. Behar M, Barken D, Werner SL, Hoffmann A The Dynamics of Signaling as a Pharmacological Target Cell 2013; 155: 448-461.
3. Hu JZ, Sealfon SC, Hayot F, Jayaprakash C, Kumar M, Pendleton AC, Ganee A, Fernandez-Sesma A, Moran TM, Wetmur JG Chromosome-specific and noisy IFNB1 transcription in individual virus-infected human primary dendritic cells Nucleic Acids Research 2007; 35: 5232-5241.
4. Apostolou E, Thanos D Virus infection induces NF-kappa B-dependent interchromosomal associations mediating monoallelic IFN-beta gene expression Cell 2008; 134: 85-96.
5. Gillespie DT Stochastic simulation of chemical kinetics Annual review of physical chemistry 2007; 58: 35-55.
6. Rand U, Rinas M, Schwerk J, Nohren G, Linnes M, Kroger A, Flossdorf M, Kaly-Kullai K, Hauser H, Hofer T *et al* Multi-layered stochasticity and paracrine signal propagation shape the type-I interferon response Molecular Systems Biology 2012; 8.
7. Zhao MW, Zhang JW, Phatnani H, Scheu S, Maniatis T Stochastic Expression of the Interferon-beta Gene Plos Biology 2012; 10.
8. Lichty BD, Power AT, Stojdl DF, Bell JC Vesicular stomatitis virus: re-inventing the bullet Trends In Molecular Medicine 2004; 10: 210-216.
9. Wilkins C, Gale M Recognition of viruses by cytoplasmic sensors Current Opinion In Immunology 2010; 22: 41-47.
10. Kawai T, Takahashi K, Sato S, Coban C, Kumar H, Kato H, Ishii KJ, Takeuchi O, Akira S IPS-1, an adaptor triggering RIG-I- and Mda5-mediated type I interferon induction Nature immunology

2005; 6: 981-988.
11. Xu LG, Wang YY, Han KJ, Li LY, Zhai ZH, Shu HB VISA is an adapter protein required for virus-triggered IFN-beta signaling Molecular Cell 2005; 19: 727-740.
12. Ajibade AA, Wang HY, Wang RF Cell type-specific function of TAK1 in innate immune signaling Trends In Immunology 2013; 34: 307-316.
13. Gupta S, Campbell D, Derijard B, Davis RJ Transcription factor ATF2 regulation by the JNK signal transduction pathway Science 1995; 267: 389-393.
14. Raingeaud J, Whitmarsh AJ, Barrett T, Derijard B, Davis RJ MKK3- and MKK6-regulated gene expression is mediated by the p38 mitogen-activated protein kinase signal transduction pathway Mol Cell Biol 1996; 16: 1247-1255.
15. Fitzgerald KA, McWhirter SM, Faia KL, Rowe DC, Latz E, Golenbock DT, Coyle AJ, Liao SM, Maniatis T IKK epsilon and TBK1 are essential components of the IRF3 signaling pathway Nature Immunology 2003; 4: 491-496.
16. Honda K, Takaoka A, Taniguchi T Type I inteferon gene induction by the interferon regulatory factor family of transcription factors Immunity 2006; 25: 349-360.
17. Tamura T, Yanai H, Savitsky D, Taniguchi T The IRF family transcription factors in immunity and oncogenesis Annual review of immunology 2008; 26: 535-584.
18. Schneider WM, Chevillotte MD, Rice CM Interferon-stimulated genes: a complex web of host defenses Annual review of immunology 2014; 32: 513-545.
19. Morales DJ, Lenschow DJ The Antiviral Activities of ISG15 Journal Of Molecular Biology 2013; 425: 4995-5008.
20. Kim MJ, Hwang SY, Imaizumi T, Yoo JY Negative feedback regulation of RIG-1-mediated antiviral signaling by interferon-induced ISG15 conjugation Journal Of Virology 2008; 82: 1474-1483.
21. Seo JY, Yaneva R, Cresswell P Viperin: A Multifunctional, Interferon-Inducible Protein that Regulates Virus Replication Cell Host & Microbe 2011; 10: 534-539.
22. Haller O, Kochs G Human MxA Protein: An Interferon-Induced Dynamin-Like GTPase with Broad Antiviral Activity Journal Of Interferon And Cytokine Research 2011; 31: 79-87.
23. Fensterl V, Sen GC The ISG56/IFIT1 Gene Family Journal Of Interferon And Cytokine Research 2011; 31: 71-78.
24. Shalek AK, Satija R, Shuga J, Trombetta JJ, Gennert D, Lu DN, Chen PL, Gertner RS, Gaublomme JT, Yosef N *et al* Single-cell RNA-seq reveals dynamic paracrine control of cellular variation Nature 2014; 510: 363-+.
25. Kreuz LE, Levy AH Physical Properties Of Chick Interferon Journal of bacteriology 1965; 89: 462-469.
