# Supplementary figures and images for: Integrated modeling and analysis of intracellular and intercellular mechanisms in shaping the interferon response to viral infection
Source: PLoS One. 2017 Oct 11;12(10):e0186105. doi: 10.1371/journal.pone.0186105 (PMC5636135; doi:10.1371/journal.pone.0186105)

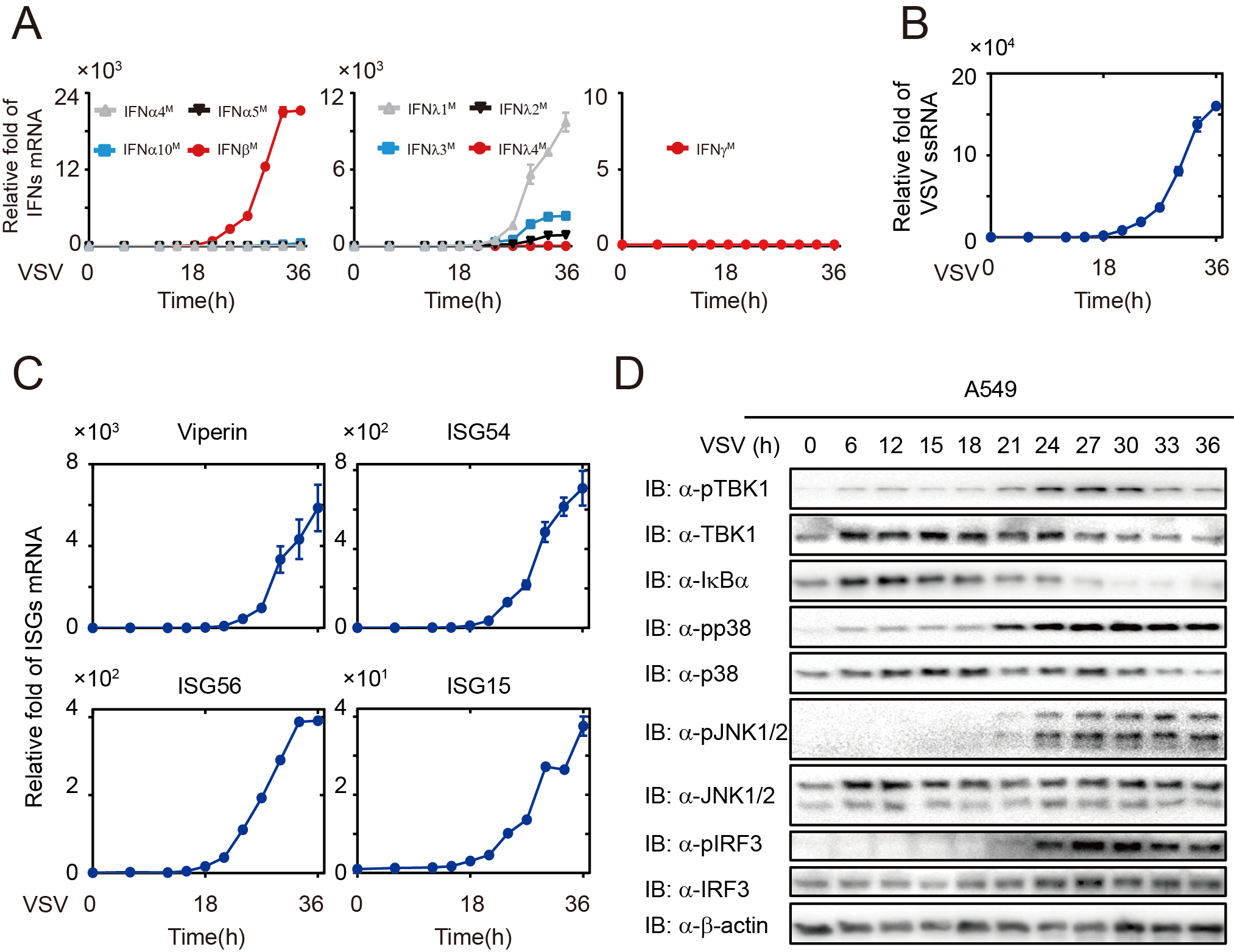

Supplement: S4 Appendix — (A) Time-course of type I, II and III IFNs expression in A549 cells infected with VSV at a MOI of 0.05. (B-C) Real-time PCR analysis of VSV ssRNA, Viperin, ISG54, ISG56 and ISG15 mRNA in A549 cells infected with VSV at a MOI of 0.05 at indicated time points. (D) Cell lysates of A549 cells were collected at indicated time after VSV (MOI = 0.05) treatment by immunoblotting with the indicated antibodies. Data in these figures were presented as the mean ± SD of three independent experiments. (TIF) [file pone.0186105.s004.tif]

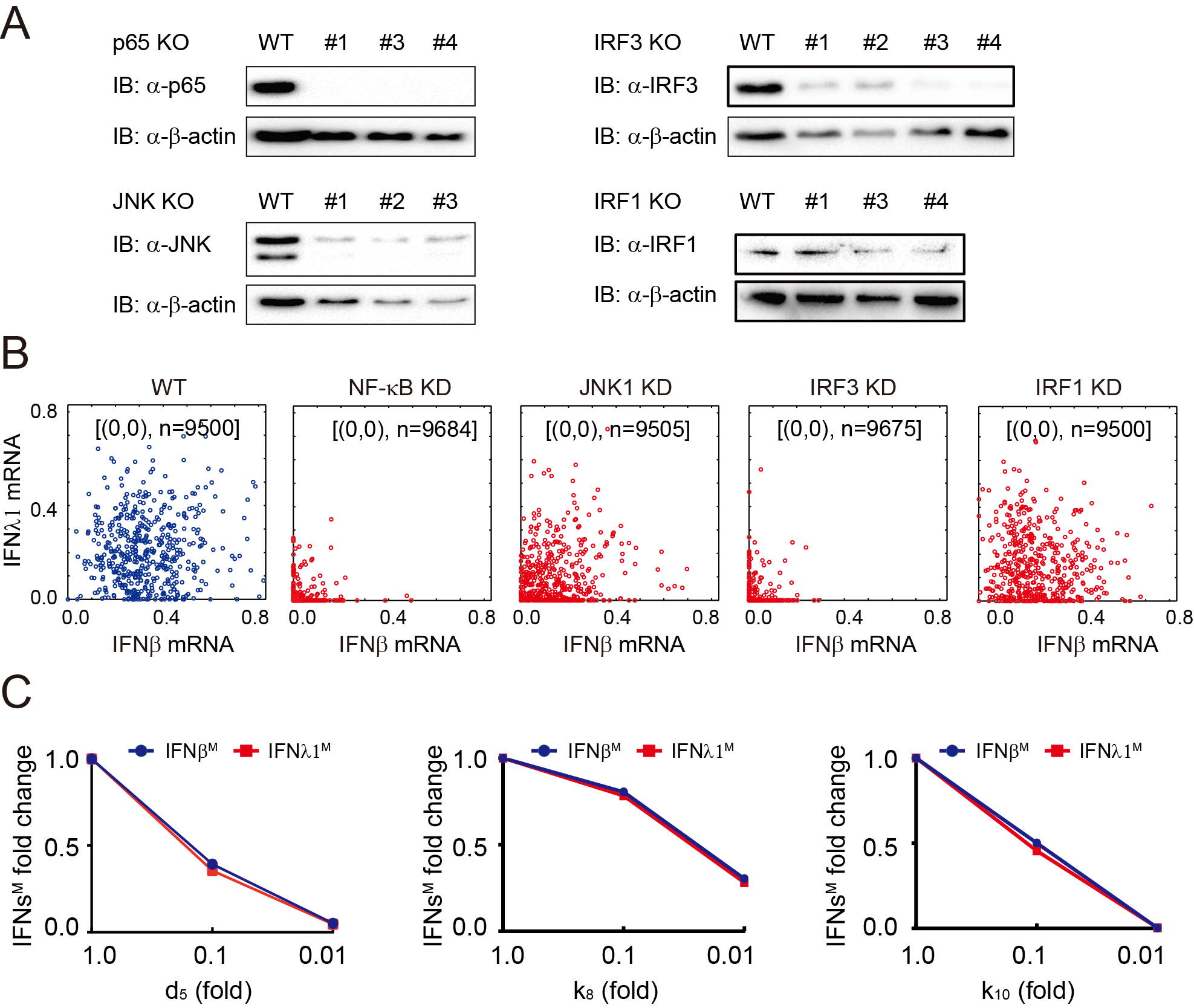

Supplement: S5 Appendix — (A) KO efficiency of p65-/-, JNK1-/-, IRF3-/- and IRF1-/- A549 cell lines by CRISPR/Cas9 technology. (B) The scatters analysis of IFNsM (t = 18h) under specified conditions. Each panel includes 10,000 simulations. (C) The change of NF-κB (left), JNK1 (middle) or IRF3 (right) activation rate (d5, k8 or k10) had similar effect on IFNβM and IFNλ1M. The blue circle and red square indicates IFNβM and IFNλ1M fold change respectively. (TIF) [file pone.0186105.s005.tif]

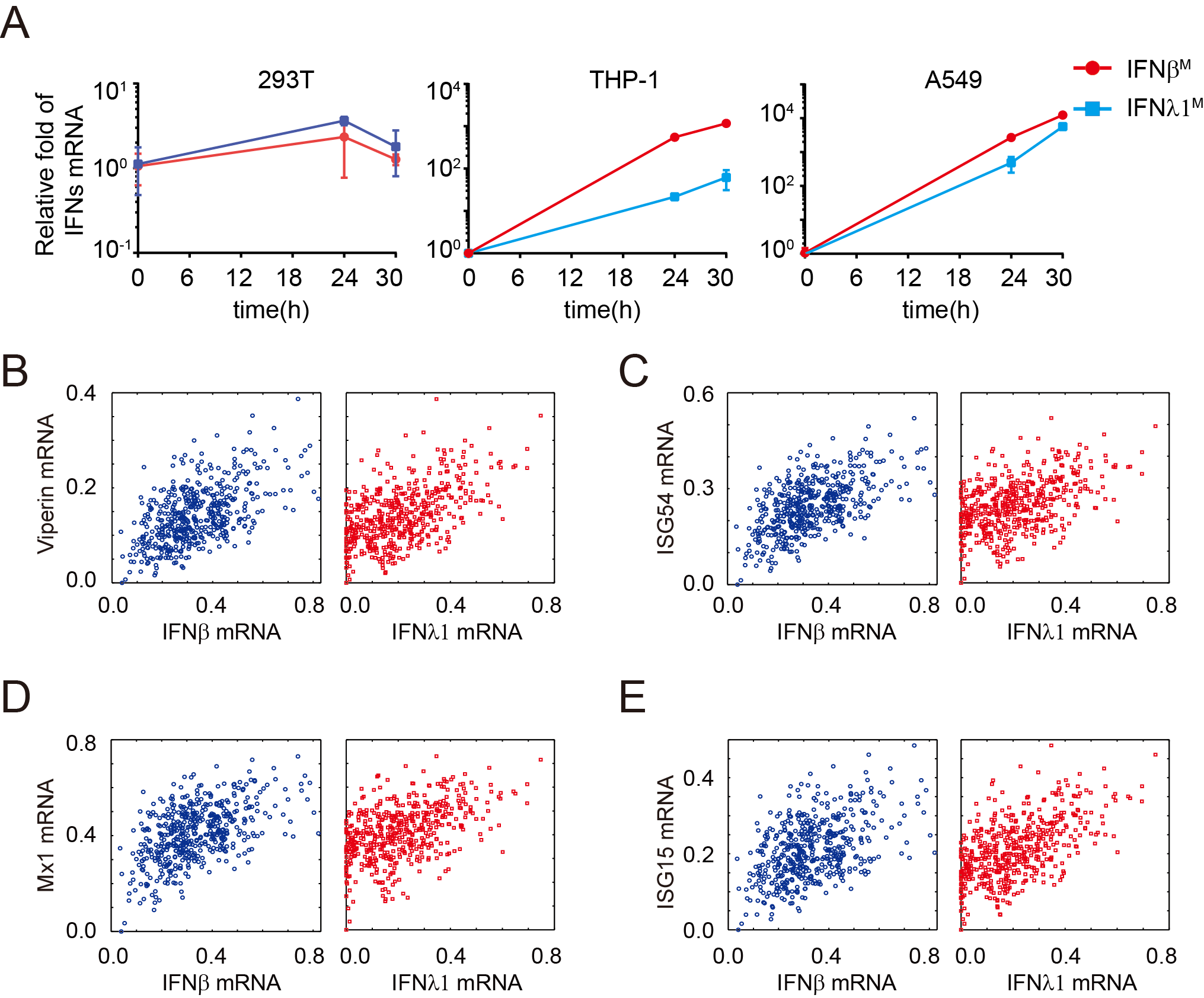

Supplement: S6 Appendix — (A) Real-time PCR analysis of IFNβ and IFNλ1 mRNA (IFNβM and IFNλ1M, respectively) in 293T, THP-1 and A549 cells infected with VSV at a MOI of 0.05. (B-E) The correlation between ISGsM (B for Viperin, C for ISG54, D for Mx1 and E for ISG15) and IFNβM (left) and IFNλ1M (right) by scatter analysis in early infected cells (n = 500, t = 18h). The squares of the Pearson correlation coefficients (r2) between ViperinM, ISG54M, Mx1M, ISG15M and IFNβM are 0.31, 0.34, 0.28 and 0.33, respectively. In addition, r2 between ViperinM, ISG54M, Mx1M, ISG15M and IFNλ1M are 0.29, 0.22, 0.22 and 0.21, respectively. The P values of all panels are less than 0.0001. (TIF) [file pone.0186105.s006.tif]
